# A retrospective study of total hip arthroplasty

**DOI:** 10.4103/0019-5413.30528

**Published:** 2007

**Authors:** RC Siwach, Virender Singh Kadyan, SS Sangwan, Rajiv Gupta

**Affiliations:** Department of Orthopedics, Pt. B.D. Sharma PGIMS, Rohtak - 124 001, Haryana, India

**Keywords:** Total hip arthroplasty

## Abstract

**Aim:**

To evaluate the functional and radiological outcome of primary total hip replacement (THR) using modular total hip system at 2-10 years follow-up.

**Materials and Methods:**

The cohort comprised 100 operated cases for total hip replacement using modular hip system, with an average follow-up of 6.02 years ranging from 2-10 years. In 61 cases cemented THR, in 36 cases hybrid and in three cases uncemented THR was done. Harris hip score was used for clinical evaluation. Osteolysis was recorded in three acetabular zones described by DeLee and Charnley and the seven femoral zones described by Gruen *et al*.

**Results:**

The average age at operation was 52.46±9.58 years. Mean follow-up duration was 6.02 years ranging from 2-10 years. Four patients died due to causes unrelated to surgery. At the last follow-up mean Harris Hip score was 83.5. Radiolucent lines were present in 39(39%) acetabular and 32 (32%) femoral components. Osteolysis was most common in Zone 7 of the femoral and Zone II and III of the acetabular component. Eight hips have been revised, five for aseptic loosening as proved by negative culture at revision and three hips for posttraumatic periprosthetic femoral fracture. One girdle stone resection was done for deep infection. Out of 96 hips available at latest follow-up, 87 primary arthroplasties were intact and functioning well.

**Conclusion:**

The results of our study support the continued use of the modular hip system. The acetabular loosening was more common than femoral in our study.

Evaluation of the long-term outcomes of an operative procedure is important to determine the durability of the procedures like total hip replacement (THR). It provides a mean for comparison of results which may give a lead to any changes in operative technique, implant design, type of joint, that occurs over time. Aseptic femoral and acetabular loosening have emerged as the most serious long-term complication of THR and the most common indication for revision.^1^ Periprosthetic fractures of the femur can be a difficult problem to manage.^2^ The purpose of the present study was to evaluate the 2-10 year results of total hip arthroplasty using modular total hip system done in our institute.

## MATERIALS AND METHODS

In this retrospective study the cohort comprised 100 patients. Fifty-two males and 48 females, who were operated for total hip replacement between 1993 and 2003, were evaluated.

Twenty-six patients had idiopathic avascular necrosis (Ficat Type 3 and 4), 17 patients had rheumatoid arthritis, six had ankylosing spondylitis while 16 patients had osteoarthrosis (nine were of primary OA and seven posttraumatic secondary OA). Thirteen patients were of failed osteosynthesis following fracture neck of femur (seven fixed with two leg screws, three with three leg screws and three where DHS); 18 patients of fracture neck of femur were operated for primary THR while four patients of failed hemiarthroplasty were also operated.

Each case was subjected to detailed history regarding age at operation, sex, clinical diagnosis, indication of surgery, unilateral or bilateral THR, type of replacement, duration after replacement, any associated co-morbid condition.

**Figure 1:**
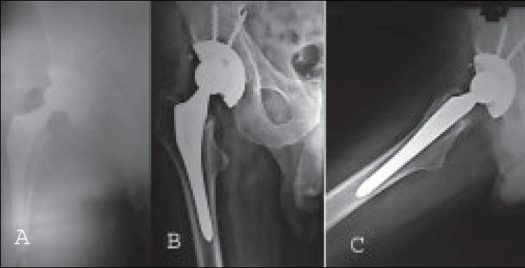
Immediate postoperative Xrays (a) of a 42-year-old male with uncemented THR with well fixed prosthesis. X-rays AP and lateral views (b and c) at five-year follow-up show no osteolysis

All cases were operated using Hardinge lateral hip approach by a single surgeon. Patients were placed supine with hip at the edge of table. Posteriorly directed lazy-J incision was made centered over the greater trochanter. Fascia lata was incised in the line of skin incision. Tensor fasciae latae was retracted anteriorly and gluteus maximus posteriorly. Gluteus medius tendon was incised obliquely across the trochanter leaving posterior half attached to trochanter. Proximally, the incision was carried in the line of fibers of the gluteus medius at the junction of the middle and posterior third. Distally, incision was carried anteriorly in the line of the vastus lareralis fibers. Anterior joint capsule was exposed and incised. Hip was dislocated by abduction and external rotation. Femoral neck was osteotomized at predetermined level as indicated by preoperative X-ray templating of hip. Femoral canal preparation was done. Chosen femoral component was implanted in 10-15 degree anteversion. Acetabular preparation was done and cup was implanted with the aim of 40-50 degree abduction and 10-15 degree anteversion. In 61 cases cemented, in three cases uncemented [Figure 1] and in 36 cases hybrid modular hip replacement was done depending upon age, activity levels, bone stock of patient. We used double-tapered polished straight stem and 28 mm modular head. In all cases Long posterior wall (LPW) High dinsity polyethylene (HDP) cup was used. We used manual mixing and finger packing of cement after inserting a bone piece as medullary plug. Sixty-eight (68%) cases were operated under combined spinal epidural anesthesia, 22 (22%) under spinal anesthesia and 10 (10%) were performed under general anesthesia.

Intravenous antibiotic (cefotaxim 1 gm and amikacin 500 mg) prophylaxis was started prior to surgery, with completion of first dose one hour before incision and it was continued seven days postoperatively. No DVT prophylaxis was used as we mobilized our patients very early. For the first two postoperative days the patients were encouraged to do in-bed exercises. The ambulation was started on the third postoperative day with walking frame. Stitches were removed after 10-14 days. Patients were discharged after proper gait training and were called for follow-up monthly for the first three months and then six-monthly. The same protocol was used for all patients. We used indomethacin 75 mg daily for six weeks postoperatively prophylactically against heterotropic ossification in all patients.

### Functional evaluation

Evaluation of functional / clinical outcome was done using Harris hip scoring system.^3^

### Radiographic evaluation

Radiographic evaluation was done by recording radiolucent lines between the cement and bone as seen on anteroposterior and lateral views of the operated hip, in three acetabular zones described by DeLee and Charnley^4^ and the seven femoral zones delineated by Gruen *et al*.^5^,^6^ We tried to evaluate loosening in a consistent manner at each six-monthly follow-up interval [Table 1].

**Table 1:** Gruen, MC Neice and Amstutz criteria of femoral component loosening^6^

Roentgenographic changes diagnostic of loosening of femoral component in total hip arthroplasty.
Following is the list of roentgenographic changes in the stem and the cement about it, suggestive or diagnostic of loosening of femoral component. Radiolucency between the superolateral one-third of the stem and adjacent cement mantle indicating debonding of stem from cement and possible early deformation of the stem. Radiolucency between the cement mantle and surrounding bone. Subsidence of the entire cement mantle and stem resulting in a more distal position of the collar or platform in relation to the proximal surface of the cement and the femoral neck. Change of the femoral stem into a more varus position. Areas of rarefaction or fragmentation of the cement, especially between the superomedial aspect of the stem and the femoral neck or in areas of their cement mantle. Fracture of cement mantle, most commonly near the tip of the stem. Deformation of the stem in the anteroposterior or lateral roentgenogram. Incomplete or complete failure (fracture) of the stem.

The mean age was 52.46±9.58 years (35 to 70 years). There were 42 patients involving the left hip and 50 with right hip and four with both sides. The most common indication was avascular necrosis (n=26) of femoral head [Table 2]. Ten patients of fracture neck femur above 60 years (physiological age) were operated for THR. Five patients had late presentation of fracture neck of the femur (more than four weeks) with osteoarthritic changes in hip while three had pathological fracture secondary to osteoporosis (n=2), metastatic deposit from carcinoma breast (n=1). Thirty-seven patients had systemic disease. Twenty-two patients were hypertensive, eight were diabetic, six had bronchial asthma and were on steroids and one patient had aplastic anemia.

**Table 2:** Criteria of acetabular component loosening^16^

The following changes in the pelvis and acetabular component can be observed in serial roentgenograms:
Absorption of bone from around part or all of the cement mantle and an increase in the width of the area of absorption, which is especially significant if more than 2 mm wide and progressive six months or more after surgery. Superior or medial migration and protrusion of the cement mantle and cup into the pelvis: also, fracture of the medial cortex of the acetabulum. Change in the angle of inclination or the degree of anteversion of the cup, indicating component migration. Wear of the cup, as indicated by a decrease in the distance between the surface of the head and the periphery of the cup. Fracture of the cup and cement (both rare). A radiolucency up to 2 mm wide with or without a surrounding fine line of density, which may develop in one or more of the three zones about the cement mass in the pelvis. As in the femur, this is produced by the dense, fibrous membrane that forms about the surface of the cement and the surrounding shell of reactive bone.

The minimum follow-up was two years with mean follow-up of 6.02 years (2-10 years).

## RESULTS

At the latest follow-up 96 cases were alive while four cases died due to natural cause after six to eight years of replacement. They were evaluated before death at their scheduled follow-up visit. Eighty had minimal or no pain, while 12 had moderate pain requiring occasional analgesics and four patients had severe pain. Eighty (80%) had good range of movement (flexion 90° or more, abduction 20° or more), 16 had mild restriction of movement (flexion <90°, abduction <20°) and four patients had severe restriction of movement.

One each of these four had deep infection, hetereotopic ossification, pathological fracture secondary to carcinoma breast and revised THR following aplastic anemia. Eighty used no walking aid, 15 used one stick and five used walking frame. No limp was present in 75 (75%) patients. Twenty-five patients having limp were either having limb length discrepancy preoperatively (n=13) that could not be corrected or due to problems related to nonunion of trochanter (n=3) or aseptic loosening of components (n=5), revision for posttraumatic periprosthetic fracture (n=3), Girdlestone excision arthroplasty (n=1). All trochanteric fractures occurred during the THR and ended in trochanteric nonunion.

In 75 (75%) cases, no lengthening or shortening was present while in 16 shortening was half inch. Five patients had shortening about one inch. In one patient shortening was more than one inch while in four (4%) cases lengthening by half inch was present.

Mean Harris hip score preoperatively was 44 (30-50) point and postoperatively 83.5 (60-96). We categorized our results as good in 75%, fair in 18% and poor in 7% cases.

**Figure 2:**
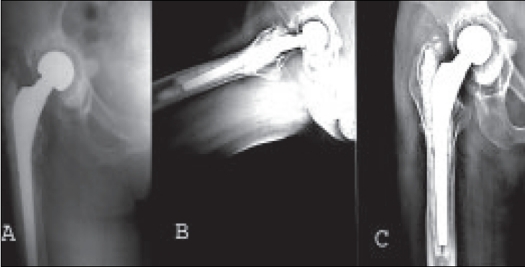
Ten years follow-up X-rays of a 65-year-old female showing osteolysis in all seven femoral and all three acetabular zones but clinically patient was asymptomatic

**Figure 3:**
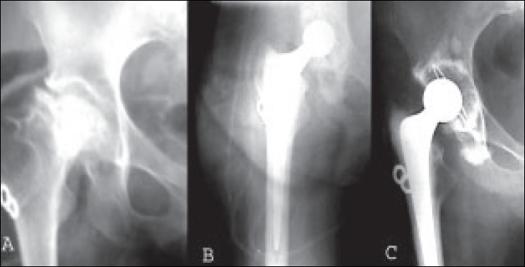
X-rays of a case of failed osteosynthesis (a) Immediate postop X-rays (b) after cemented THR. Seven years follow-up X-ray (c) showing osteolysis in Zone I and IV of femoral component and Zone I of acetabular component

Thirty-nine (39%) cases had osteolysis around the acetabular and 32 (32%) around the femoral components [Figure 2]. Ten hips (10%) had osteolysis in Zone I of the acetabulum, seven (7%) in Zone I and II, 10 (10%) in Zone II and III, nine (9%) in Zone I and III, five (5%) in Zone III. Twenty (20%) femoral components had osteolysis in Zone VII (7%) and five (5%) femoral components had osteolysis in Zone I and VII [Figure 3]. Osteolysis was most common in Gruen Zone VII of the femoral component and DeLee and Charnley Zone II and III of the acetabular component.

**Figure 4:**
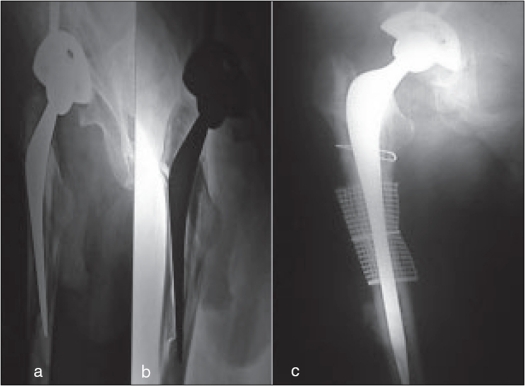
(a-c) X-rays of a patient with periprosthetic fracture six years after index surgery due to roadside accident. Femoral component revised with long stem

**Figure 5:**
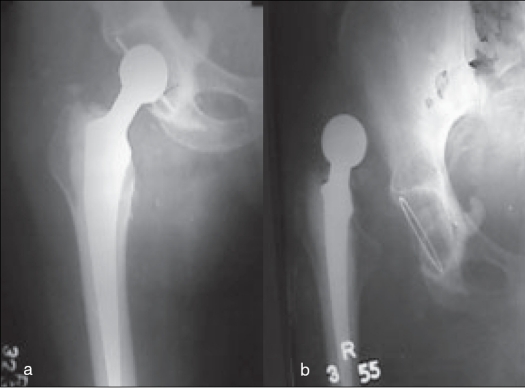
(a,b) Complication- dislocation hip

Out of 100 patients, four had superficial infection which was treated by antibiotics following culture sensitivity [Table 3]. One patient had deep infection requiring removal of implant and Girdlestone arthroplasty. Six cases had postoperative transient femoral neuropraxia due to anteriorly placed levers which recovered fully in six to 12 weeks. One foot drop occurred which recovered after three months. One patient developed heterotropic ossification which led to significant restriction of hip movements. Three male patients sustained posttraumatic periprosthetic femoral fractures, who met roadside accidents after 6 years in 2 patients [Figure 4] and after 4 years in one patient after the index procedure. They were managed by revision of the femoral component with long stem. There were five dislocations [Figure 5], two occurred during the first month of hip replacement and three occurred during the first year; all were posterior and were managed by closed reduction. There were three aseptic acetabular loosening and two femoral loosening requiring revisions. The diagnosis of aseptic loosening was made clinicoradiologically. These patients had pain on weight bearing that was relieved by rest. All had positive antalgic gait. On X-rays radiolucent zone of 2 mm or more in one (n=3) or both (n=2) components was found.

**Table 3:** Complications

	No. of cases (%)
Early	
Hematoma	4 (4)
Infection	4 (4)
Superfacial	1 (1)
Deep	
Trochanteric #	3 (3)
Splintering of shaft	1 (1)
Foot drop	1 (1)
Femoral nerve palsy	6 (6)
Late	
Heterotrophic ossification	1 (1)
Trochanteric nonunion	3 (3)
Periprosthetic fracture	3 (3)
Hip dislocation	5 (5)
Aseptic loosening	
Femoral	2(2)
Acetabular	3(3)

Eighty-seven patients have retained original implants and are functioning well. Eight patients had revision, five for aseptic loosening of components and three for periprosthetic fractures. One Girdlestone resection was done for deep infection.

## DISCUSSION

In the present era, total hip arthroplasty is not the operation of the elderly alone. Due to immense research on this subject in operative technique, technology and biomaterial, it is now very much possible to perform this operation in the younger age group patients depending upon the patient's age, activity, occupation and other social obligations.

The threshold for THR has changed; more young patients with less severe symptoms are now being offered THR. The traditional management of turning a deaf ear to patients’ complaint of hip pain until he is older is no longer accepted.^13^

The availability of modular prosthesis allows the surgeon intraoperative versatility, allowing adjustment of leg length, neck length, valgus and varus positioning of stem, as centralizer is provided in this hip assembly.^14^,^15^

Total hip replacement is performed to achieve painless, mobile, stable hip with restoration of limb length. Eighty-four per cent patients were absolutely pain-free, 12% patients had moderate pain and 4% patients had severe pain. Cupic and Zoran^7^ reported 91.3% (n = 106) patients with pain-free hip and moderate pain in 8.7%.

We achieved good range of motion in 80% patients, while 16% had mild restriction and 4% patients had severe restriction of movement. Zoran and Cupic^7^ reported 78.7% good, 18.3% mild and 3% had severe restriction in movement.

We could maintain good clinical results for 2-10 years. In 42 (42%) patients excellent roentgenographic appearance was also maintained for up to 10 years. No osteolysis was present in 68% femoral and 61% acetabular components. The functional parameters and clinical hip scores deteriorated somewhat with advancing duration of follow-up.

Osteolysis is the term used to describe periprosthetic bone loss that has been recognized as a major long-term complication of THA - cemented or cementless. Osteolysis poses two interrelated problems: a) bone loss and b) loosening of components which is often multifactorial with quality of initial fixation playing a major role in determining the long-term outcome of arthroplasty. Although the exact pathophysiological mechanism of osteolysis remains unclear, the common pathway leading to the development of osteolysis is the phagocytosis of submicron particulate debries by the macrophages, thereby triggering a cascade of events causing periprosthetic bone loss. The clinical consequences of wear debries cover a broad spectrum from radiolucencies to massive bone loss and implant failure.^8^ Concerns^9^,^10^ have been raised about rapidly increasing loosening of components as the length of follow-up increases. The incidence of aseptic loosening in our series was 5%. In the Exeter series^11^ the incidence of aseptic loosening was 5% and in the Zoran and Cupic series 2%.^7^ Loosening was more frequent in younger, more active patients. Age-related decrease in activity may lower the incidence of probable loosening in elderly patients.

During entire follow-up three patients had periprosthetic femoral fracture, managed by revision of femoral component with long stem. In our series five patients (5%) sustained dislocation. All were posterior dislocations. The etiology of dislocation after arthroplasty is multifactorial-many factors like soft tissue tension, surgical approach, patient compliance, implant position and implant design play a role. In our patients who sustained dislocation, the acetabular component was malpositioned in retroversion. In the Zoran Cupic^7^ series the dislocation rate was 1.6% whereas in the Callaghen and Albright^12^ series it was 4%.

Hip scores are convenient for rating the results of total hip arthroplasty and allow coordination of multiple parameters, including functions and pain relief. Using Harris hip score, we graded our results as good in 75% cases, fair in 18% cases and poor in 7% cases. Zoran Cupic^7^ had 91% patients classified as good, 6% as fair and 3% as poor.
